# Highly conformal combined radiotherapy with cisplatin and gemcitabine for treatment of loco-regionally advanced cervical cancer – a retrospective study

**DOI:** 10.1186/s13014-017-0938-1

**Published:** 2017-12-22

**Authors:** Nikola Cihoric, Alexandros Tsikkinis, Eugenia Vlaskou Badra, Markus Glatzer, Urban Novak, Amina Scherz, Mohamed Shelan, Ivan Soldatovic, Chittazhathu Kurian Kuruvilla Yojena, Daniel M. Aebersold, Kristina Lössl

**Affiliations:** 1 Department of Radiation Oncology, Inselspital, Bern University Hospital, University of Bern, Freiburgstrasse 15, 3010 Bern, Switzerland; 2 Department of Medical Oncology, Inselspital, Bern University Hospital, University of Bern, Bern, Switzerland; 3 Department of Radiation Oncology, Kantonsspital, St. Gallen, Switzerland

**Keywords:** Cervix cancer, Chemo-radiation, Cisplatin, Gemcitabine, Highly conformal radiotherapy

## Abstract

**Background:**

Cisplatin and gemcitabine combined with conventional radiation therapy in the treatment of cervical cancer patients results in a favorable outcome but with excess toxicity. The purpose of this study was to evaluate the toxicity profile of dual chemotherapy and highly conformal external beam radiotherapy with image guided adaptive brachytherapy.

**Methods:**

Seventeen patients with cervical carcinoma FIGO stage IB2–IIIB were treated with curative intent between 2011 and 2015. A total dose of 50.4 Gy was prescribed to the elective pelvic nodal volume. Patients with ^18^FDG-PET/CT positive lymph nodes (*n* = 15; 83.3%) received an additional boost to a total dose of 62 Gy. Chemotherapy prescription goals were: concomitant during 5 weeks of external beam radiotherapy (EBRT) 40 mg/m2 cisplatin and 125 mg/m^2^ gemcitabine, followed by adjuvant chemotherapy from week 10 (2 cycles 50 mg/m^2^ cisplatin and 1000 mg/m^2^ gemcitabine). EBRT was followed by 3–4 fractions (6 Gy per fraction) of intrauterine image guided adaptive brachytherapy. Toxicities were graded according to the common terminology criteria for adverse events (CTCAE v 4.0).

**Results:**

One (6%) patient developed acute grade 3 diarrhea. We did not record any other acute or late gastrointestinal or urogenital toxicity higher that grade 3. Most common acute hematological toxicity was anemia grade 2 recorded in 10 (59%) patients. There was only one case of grade 3 neutropenia (6%). The number of patients that received the complete chemotherapy regimen was gradually declining during the course of therapy. From week 2 to 5, gemcitabine was omitted in 4 (24%),7 (41%), 8 (47%), and 12 (71%) patients respectively, similarly, cisplatin was omitted in 2 (12%),3 (18%),1 (6%) and 7 (41%) patients respectively. Adjuvant chemotherapy was omitted in 8 patients (47%). During a median follow-up time of 20 months (5 to 63 months) 6 (35%) patients developed disease relapse with 5 (29%) of them in the form of systemic disease.

**Conclusions:**

In contrast to previous findings cisplatin and gemcitabine in combination with highly conformal radiation therapy seems to have an acceptable toxicity profile. Further studies are needed to determine the optimal dosage of the proposed therapy concept.

## Background

The standard therapeutic approach for patients with loco-regionally advanced cervical cancer is combined radio-chemotherapy (RCT). Several large prospective randomized clinical trials have shown that the combination of radiotherapy with cisplatin based chemotherapy prolongs the disease-free survival while reducing mortality. Even though the outcome of RCT is better than that of radiation alone, the combined regimen is associated with a higher incidence of side effects while distant disease control and overall survival remains unsatisfactory [1, 2]. Between 20 and 40% of patients treated with conventional radiotherapy will relapse loco-regionally, not only outside of the treatment volume but also within [3].

At the same time, rapid advancements in the radiation delivery technology and techniques as well as cancer imaging were achieved. These changes are well mirrored in a significantly improved therapeutic ratio of intensity modulated radiotherapy (IMRT) for various tumor entities, including cervical cancer [4]. The incorporation of multimodal imaging, especially in patients with cervical cancer, enhances the detection of nodal and/or systemic disease while allowing better patient selection and definition of loco-regional disease [5, 6]. It is now possible to treat lymph node metastases using dose escalated radiotherapy without significant toxicity and modern image guided adaptive brachytherapy (IGABT) results in local control higher than 90% [7].

Nevertheless and despite of all the advances made possible, systemic disease recurrence after treatment remains a problem. The challenge of reducing disease recurrence is well known and the potential answer to it may indeed lie in the addition of a further chemotherapy agent to the current standard of care with single agent cisplatin.

Based on the improved treatment results of doublet chemotherapy, published by Dueñas-Gonzáles et al. [8], and excellent toxicity profile achieved with highly conformal radiotherapy, we used a combination of cisplatin and gemcitabine as standard concomitant and adjuvant chemotherapeutic combination for patients with loco-regionally advanced cervical cancer in 2011.

The goal of this study was to evaluate early toxicity of doublet concomitant chemotherapy (cisplatin and gemcitabine) followed by two adjuvant cycles, with dose escalated volumetric modulated external beam radiotherapy followed by intrauterine IGABT.

## Methods

### Patients

Patients with histologically confirmed cervical cancer, FIGO stage IBI to IVA, treated with IMRT and concomitant combined chemotherapy with cisplatin and gemcitabine at the Bern University Hospital Department of Radiation Oncology were included in this retrospective study according to the institutional ethical standards.

We evaluated all medical and radiotherapy records, pretreatment and follow-up data as well as radiographic images of 17 patients treated between May 2011 and December 2015. Patient characteristics are summarized in Table 1.

**Table 1:** Patient Characteristic

	No.	%
FIGO Stage		
IB	3	16.7%
IB1	7	38.9%
IB2	1	5.6%
IIA	1	5.6%
IIB	3	16.7%
IIIA	2	11.1%
IIIB	1	5.6%
Nodal Status		
pN1	7	38.9%
cN1	9	50.0%
cN0	2	11.1%
Tumor Size		
< 4 cm	3	16.7%
> 4 cm	15	83.3%
Number of metastatic lymph nodes		
0	2	11.1%
1	6	33.3%
2	4	22.2%
3	1	5.6%
> 3	5	27.8%
Presence of metastatic para-aortic lymph nodes		
No	13	72.2%
Yes	5	27.8%
Tumor hystological type		
Squamous cell carcinoma	13	72.2%
Adenocarcinoma	5	27.8%
Tumor grade		
G1	0	0.0%
G2	13	72.2%
G3	2	11.1%
Data not available	3	16.7%

### Staging and treatment

All patients underwent a pretreatment staging workup as defined by institutional standards (medical history, general physical and gynecological examination under anesthesia, digital rectal examination, tumor biopsy, comprehensive laboratory blood analysis, rectoscopy and cystoscopy). Tumor staging was defined according to the International Federation of Obstetrics and Gynecology (FIGO) and the TNM-UICC system (7th edition). Further staging and radiotherapy as well as surgical treatment details are described in detail in our previously published work [9, 10].

### Radiotherapy

Contouring: Image sets acquired by CT, diagnostic ^18^FDG-PET/CT and MRI were imported into the Eclipse Planning System (Varian Medical System, Palo Alto, CA). The gross tumor volume of the cervix (GTVc) was defined as the visible macroscopic tumor based on all of the available clinical and imaging data. Clinical target volume for primary tumor area (CTVc) encompassed GTVc, uterus, parametria and the upper third of the vagina. In cases of vaginal involvement, CTVc was expanded 2 cm into the vagina caudally of the tumor. The planning target volume of the primary tumor (PTVc) was created using anisotropic expansion, taking cervical and surrounding structure movements into consideration. The PTVc was expanded to 15 mm in the antero-dorsal direction and 10 mm in the lateral direction. PTVc was manually corrected when needed. In the dorsal direction PTVc margin extended maximally to the posterior rectal wall and maximally 1.5 cm anteriorly into the bladder [11].

Dose Prescription: In the first phase PTVc was irradiated with a total dose of 50.4 Gy. After 45 Gy a control MRI was performed to evaluate tumor response and measure tumor size. In cases where the remaining tumor was larger than 4 cm in diameter, an additional EBRT boost of 9 Gy was administered to the PTVc. Otherwise, for tumors smaller than 4 cm in diameter, the PTVc was irradiated with an EBRT boost of 5.4 Gy. The single dose used was always 1.8 Gy.

Dose Constraints: Dose constraints for organs at risk were standardized as follows: 60% of rectal volume should receive no more than 50 Gy, 35% of bowel volume should receive no more than 35Gy, 50% of bladder volume should receive no more than 50 Gy and 10% of the femoral heads volume should receive no more than 50 Gy.

Brachytherapy: EBRT was followed by a HDR boost to the primary tumor. We used a microSelectron® HDRB Unit and a Vienna Ring CT-MRI Applicator Set. MRI images were evaluated together with our radiology department to define the high and intermediate risk areas. Afterwards, we reconstructed the high risk and intermediate risk areas detected in the MRI images on our planning CT images [12, 13]. Brachytherapy consisted of a total dose of 18 Gy delivered in 4 weekly fractions, with a single dose of 6 Gy.

### Chemotherapy

All patients were scheduled to receive cisplatin 40 mg/m^2^ by intravenous infusion over 60 min followed by gemcitabine 125 mg/m^2^ administered by intravenous infusion over 30 min once a week for 6 weeks, combined with radiation treatment on days 1–5 over 6 weeks respectively. Immediately after completion of the chemo-radiotherapy schedule, patients underwent brachytherapy (BCT) in week 7, followed by two weeks of rest. Thereafter patients underwent two consecutive 21-day cycles of adjuvant chemotherapy with cisplatin 50 mg/m^2^ on day 1 and gemcitabine 1000 mg/m^2^ on days 1 and 8. The treatment schema was based on rules defined by Dueñas-González et al. [8].

### Dose reduction rules

According to the internal standards of the University Hospital of Bern, multiple variables were controlled and were required to be met to fulfill the necessary safety requirements prior to each chemotherapy cycle: ECOG 0–2, thrombocytes >100 G/l, leucocytes >3 G/L or neutrophils >1.0 G/l, neurotoxicity less than grade 3 according to CTCAE v4 and calculated creatinine clearance of at least 60 ml/min (by Cockcroft-Gault). These factors were evaluated weekly by the responsible oncologists.

In cases of hematological toxicity, or reduced general condition, gemcitabine was reduced or omitted, in cases of neurotoxicity, or renal insufficiency cisplatin was reduced or omitted. Erythrocyte transfusion limit was Hb ≤ 80 g/l. In cases of fever and infection, chemotherapy dose was delayed.

### Supportive therapy for prevention of nausea, kidney damage, hematologic toxicity

Concomitant treatment for the prevention of nausea were aprepitant (125 mg on the first day of chemotherapy and 80 mg on the second and third day), methylprednisone (125 mg on the first day) and dexamethasone (4 mg from the second to the fourth day).

In order to prevent kidney damage, hydration treatment was provided by intravenous infusion of 1000 ml fluid before and after cisplatin application (0.9% NaCl before and Glucosaline 2:1 after cisplatin +10 mm KCl and 8 mm MgCl2). Sodium Chloride and Glucose Solution for Infusion 0.18% and 4%.

Erythrocyte transfusions were given for symptomatic anemia or hemoglobin levels ≤80 g/l. Platelet transfusions were given for Tc < 20 G/l with bleeding or fever, otherwise if <10G/l. Filgrastim was used to prevent severe neutropenia.

Statistical methodology: Results are presented as count (percent) or median (minimum-maximum). The Kaplan-Meier curve was used for disease-free survival (DFS) analysis. DFS was measured from the first day of therapy to the first, clinical or radiological or metabolic sign of disease.

## Results

### Treatment related toxicities

**Fig. 1:**
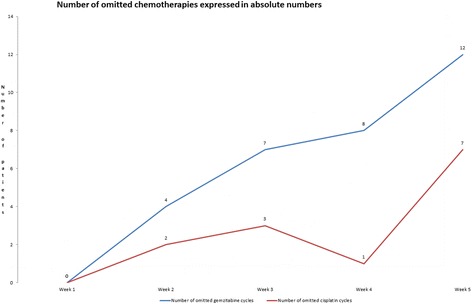
Number of omitted chemotherapies expressed in absolute numbers

There were no significant pauses or omissions of radiotherapy. The number of omitted chemotherapy applications during the concomitant part is presented in Fig. 1. Adjuvant combined gemcitabine / cisplatin chemotherapy was given in 10 (58.8%) and 9 (52.9%) patients in the first and second adjuvant cycle respectively.

Treatment related toxicities were recorded in the majority of patients (*n* = 15; 88.2%), however we did not record any kind of life threatening toxicities (≥grade 4 CTCAE v4.0) and there were no treatment related deaths recorded.

### Acute toxicities

Diarrhea was recorded in 9 (52.9%) patients, of those 4 (23.5%) were grade 1, 4 (23.5%) were grade 2 and 1 (5.9%) was grade 3. Nausea was noted in 5 (29.4%) patients, of those grade 1 in 2 (11.8%), grade 2 in 1 (5.9%) and grade 3 in 2 (11.8%) patients. Acute cystitis was noted in 4 (23.5%) patients, of those 3 (17.6%) grade 1 and 1 (5.9%) grade 2. Vaginitis emerged in 4 (23.5%) patients during treatment, of those 3 (17.6%) patients had grade 1 and 1 (5.9%) grade 2. No other gastrointestinal or genitourinary toxicities were reported.

Other therapy related acute toxicities were recorded as follows: 1 patient (5.9%) developed a lymphocele with consequent ureteral obstruction that required a nephrostomy and surgical intervention afterwards (as a direct consequence of prior lymphadenectomy). Two (11.8%) patients developed polyneuropathy grade 2. Furthermore, one (5.9%) patient developed intraabdominal fat tissue necrosis that was treated conservatively and one (5.9%) patient developed low back and pelvic pain grade 2. A commonly described adverse event was fatigue (*n* = 8, 47.1%), grade 2 in seven (41%) patients and grade 3 in 1 (6%) patient.

**Fig. 2:**
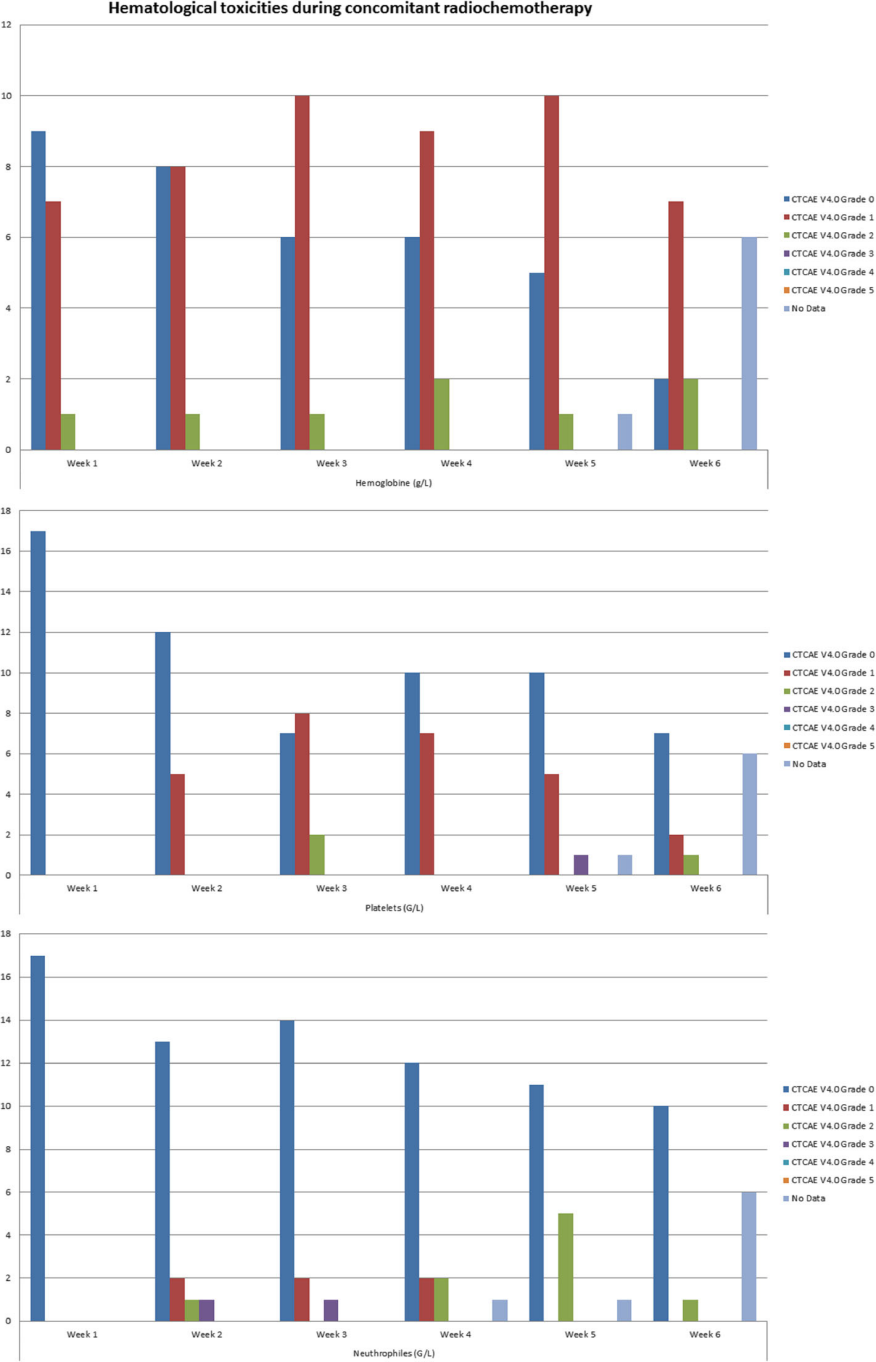
Hematological toxicities during concomitant radio-chemotherapy

Acute hematological toxicities during concomitant radio-chemotherapy are presented in Fig. 2 in the form of cumulative incidence.

### Chronic toxicities

Chronic gastrointestinal or urogenital toxicity was reported or diagnosed in 10 (58.8%) patients. Five (29.4%) reported upper gastrointestinal toxicities grade 1, 4(23.5%) reported cystitis grade 1, and one patient (5.9%) grade 2. Two (11.8%) patients reported vaginal dryness grade 1, one (5.9%) shortening of vagina grade 1, and two (11.8%) patients reported grade 3 toxicities in the form of a vaginal stenosis.

## Survival

Within a median follow-up of 25 months (range 5 to 62 months) 7 (41.2%) patients developed disease recurrence. One (5.9%) patient developed an isolated local relapse and one (5.9%) local relapse with simultaneous systemic metastatic disease. Two (11.8%) patients developed regional nodal relapse with one of them also developing systemic metastases. In total 6 (35.3%) patients developed systemic metastases. One of them died one year after diagnosis of systemic disease and two of them died years after diagnosis of systemic disease. Three patients with metastatic disease were alive at the time of our last follow up.

One (5.9%) patient developed lung metastases (detected by ^18^FDG-PET/CT) six months after initial therapy, treated with wedge resection. The patient was disease free at the point of our last follow-up visit at 62 months after initial therapy and 56 months after resection of the metastasis.

One (5.9%) patient developed a solitary liver metastasis 13 months after initial treatment. She was surgically treated. After 4 months, this patient showed a new FDG-active lesion within the former tumor bed in the liver for which she received stereotactic body irradiation. The patient was alive and in good health, without any signs of tumor during our last follow up 60 months after initial treatment and 36 months after treatment for metastatic disease.

**Fig. 3:**
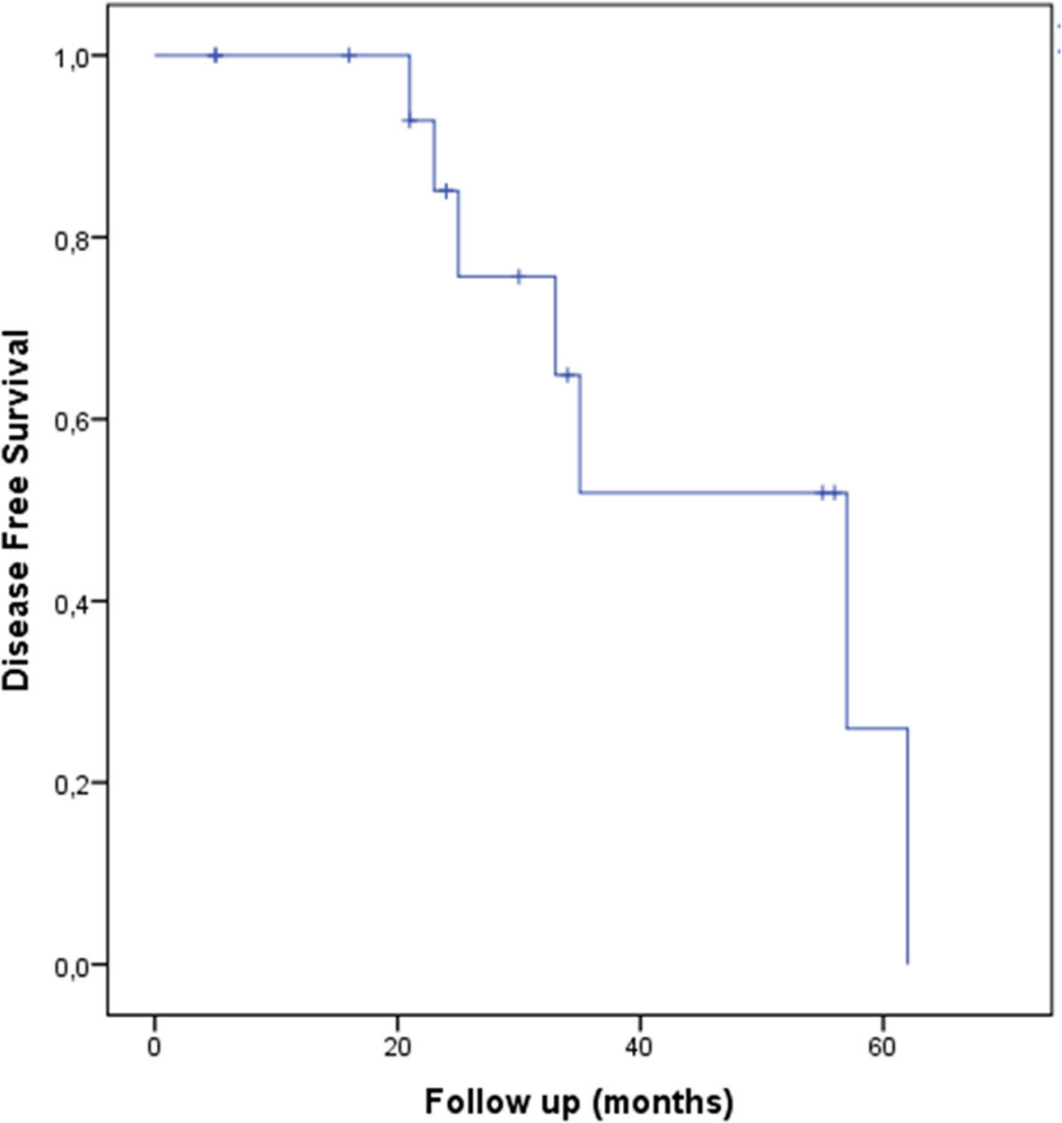
Kaplan-Meyer Curve for Disease Free Survival

Median disease free survival was 57 months (range: 32.9–81.3 months). Kaplan-Meier curve for disease free survival is shown in Fig. 3.

## Discussion

Radiotherapy alone in the treatment of loco-regionally advanced cervical cancer has unsatisfactory results. With the aim of enhancing treatment outcome several trials were designed and conducted by the end of the last century. The combined results of those pivotal trials were evaluated in a meta-analysis conducted on individual patient data by Vale C. et al. The addition of chemotherapy reduced both, local and distant recurrence and resulted in a 6% improvement in the 5-year survival compared with radiotherapy alone [14].

A combination of two chemotherapy agents may have an additive effect. Besides cisplatin, gemcitabine has shown a radio-sensitizing effect in vitro as well as in vivo [15–18]. Burnett A.F. described a 41% overall response rate with an acceptable toxicity profile [19]. Dueñas- González et al. (2001) explored the neo-adjuvant application of cisplatin / gemcitabine chemotherapy. The most common hematological toxicity was granulocytopenia grade 3 and 4 in 13.8% and 3.4% of patients respectively [20]. Further analysis showed that the neo-adjuvant approach is at least as effective as standard concurrent cisplatin based radio-chemotherapy [21].

Zarbá JJ et al. conducted a phase I/II study of weekly cisplatin and gemcitabine on 36 patients and found that the maximum-tolerated dose for Gemcitabine was 150 mg/m^2^ [22]. Further Umanzor J et al. conducted a phase II trial on 23 patients resulting in an acceptable toxicity profile with only one case of grade 3 neutropenia, no grade 4 hematological toxicities and no unusual late radiation toxicities [23].

Those early data were supported by a pivotal trial conducted by Dueñas-González. Investigators compared concurrent gemcitabine plus cisplatin and radiation followed by adjuvant gemcitabine and cisplatin with concurrent cisplatin and radiation in patients with stages IIB to IVA cervical cancer. Treatment resulted in an improved progression free survival (PFS) at 3 years (74.4% vs 65.0% *p* = 0.029), improved overall PFS (log-rank *p* = 0.0227; hazard ratio [HR], 0.68; 95% CI, 0.49–0.95) and overall survival (OS) (log-rank *p* = 0.0224; HR, 0.68; 95% CI, 0.49–0.95) and distant failure rate (8.1% vs. 16.4%, *p* = 0.005). This success was accompanied by higher grade 3 and 4 toxicities in the experimental arm (86.5% vs 46.3%; *p* < 0.001) [8].

Compared with previous trials our study has several distinct characteristics regarding methodology and patients. First of all, our patients were treated with highly conformal radiotherapy while previous studies utilized conventional EBRT. This is the possible reason for the lower incidence of grade 3 hematological toxicities. It has been shown that IMTR is better compared to two-dimensional (2D) and three-dimensional (3D) techniques, in terms of both bone marrow sparing and lower incidence of toxicity [24, 25].

Our treatment results are comparable with those published in literature. Loco-regional control is better than in previous trials that have used the cisplatin-gemcitabine combination. This may be attributed to the utilization of intrauterine adaptive image guided brachytherapy, rather than to the addition of chemotherapy. Local failure was an issue in the Dueñas-González trial although the local failure rate did not differ significantly in the two arms of the study (11.2% vs. 16.4%, *p* = 0.097) [8]. Still, disease control is problematic as 40% of patients developed systemic metastases.

Modern RT technique results in excellent local control rates. The three year local control rate exceeds 95% irrespective of tumor size [26] and reaches almost 100% if the EQD2 dose to the “high risk tumor volume” exceeds 87 Gy [7]. These facts point to the conclusion that combining chemotherapy during radiation does not produce a significant effect. However, even in the absence of visible local disease at the time of RCT, local lymph nodes may still harbor microscopic disease and macroscopic disease may also be present and remained undetected by radiological investigations such as computed tomography or MRI [27, 28]. It is possible that the administration of combined chemotherapy during EBRT can lead to the successful eradication of nodal disease.

Limitations: a major limitation of our study is its retrospective nature, and the small number of patients included in the study. Furthermore, the dose reduction or even omission of chemotherapy was not done in strict accordance to internal rules in all patients. More than 70% of all patients did not receive the prescribed chemotherapy. In addition, it was not feasible to retrieve all data on the concomitant chemotherapy, especially regarding the application of granulocyte colony-stimulating factor. As a consequence of the previously mentioned limitations we cannot draw a definitive conclusion about the safety of the proposed concept.

Despite these limitations, we believe that this study provides a unique insight on new possibilities for the treatment of patients with loco-regionally advanced cervical cancer. The presented data may serve as a starting point for sample size calculation for future prospective studies.

## Conclusions

The proposed treatment regimen seems to be promising in terms of lower toxicity than previously recorded in other studies. Further prospective studies with a higher number of patients are warranted and are necessary for the definitive confirmation of the safety and efficacy of the proposed concept.

## Abbreviations

CTCAE: Common Terminology Criteria For Adverse Events
EBRT: External Beam Radiotherapy
ECOG: Eastern Cooperative Oncology Group
FIGO: International Federation Of Gynecology And Obstetrics
IGABT: Image Guided Adaptive Brachytherapy
IMRT: Intensity Modulated Radiotherapy
PFS: Progression Free Survival
RCT: Radio-Chemotherapy
UICC: Union For International Cancer Control
